# Diversity and phylogenetic relationships among *Bartonella* strains from Thai bats

**DOI:** 10.1371/journal.pone.0181696

**Published:** 2017-07-20

**Authors:** Clifton D. McKee, Michael Y. Kosoy, Ying Bai, Lynn M. Osikowicz, Richard Franka, Amy T. Gilbert, Sumalee Boonmar, Charles E. Rupprecht, Leonard F. Peruski

**Affiliations:** 1 Division of Vector-Borne Diseases, Centers for Disease Control and Prevention, Fort Collins, CO, United States of America; 2 Department of Biology, Colorado State University, Fort Collins, CO, United States of America; 3 Division of High-Consequence Pathogens and Pathology, Centers for Disease Control and Prevention, Atlanta, GA, United States of America; 4 National Wildlife Research Center, USDA/APHIS/Wildlife Services, Fort Collins, CO, United States of America; 5 Faculty Sciences and Public Health, Rajapruk University, Nonthaburi, Thailand; 6 LYSSA LLC, Lawrenceville, GA, United States of America; 7 Center for Global Health, Centers for Disease Control and Prevention, Atlanta, GA, United States of America; University of Pretoria, SOUTH AFRICA

## Abstract

Bartonellae are phylogenetically diverse, intracellular bacteria commonly found in mammals. Previous studies have demonstrated that bats have a high prevalence and diversity of *Bartonella* infections globally. Isolates (n = 42) were obtained from five bat species in four provinces of Thailand and analyzed using sequences of the citrate synthase gene (*gltA*). Sequences clustered into seven distinct genogroups; four of these genogroups displayed similarity with *Bartonella* spp. sequences from other bats in Southeast Asia, Africa, and Eastern Europe. Thirty of the isolates representing these seven genogroups were further characterized by sequencing four additional loci (*ftsZ*, *nuoG*, *rpoB*, and ITS) to clarify their evolutionary relationships with other *Bartonella* species and to assess patterns of diversity among strains. Among the seven genogroups, there were differences in the number of sequence variants, ranging from 1–5, and the amount of nucleotide divergence, ranging from 0.035–3.9%. Overall, these seven genogroups meet the criteria for distinction as novel *Bartonella* species, with sequence divergence among genogroups ranging from 6.4–15.8%. Evidence of intra- and intercontinental phylogenetic relationships and instances of homologous recombination among *Bartonella* genogroups in related bat species were found in Thai bats.

## Introduction

Of the emerging infectious diseases in humans, most are zoonoses originating in wildlife [1,2]. Thus, surveillance and characterization of zoonotic infections is fundamental to protecting public health and understanding infectious disease ecology. Following the discovery that numerous severe viral infections are linked to bats [3], efforts to detect and understand the dynamics of viral, bacterial, and fungal zoonotic infections of bats have increased in recent years.

One genus of bacteria, *Bartonella*, is frequently found in wildlife. Bartonellae (Rhizobiales, Alphaproteobacteria) are fastidious, facultative, hemotropic bacteria that infect a variety of mammalian groups, including ungulates, rodents, carnivores, and primates [4]. Hematophagous arthropods (e.g., flies, ticks, fleas, and mites) are believed to be the primary vectors of *Bartonella* spp. infections [5,6] and studies have demonstrated the competence of a small number of vector species [7–11]. Over 30 *Bartonella* species have been named and characterized, and candidate species are being discovered as new mammal species and their ectoparasites are sampled. Among the named *Bartonella* species, approximately half have now been identified as zoonotic and can cause a wide spectrum of illnesses in people ranging from self-limiting fever to endocarditis [12–14]. Given the potential health impacts of bartonellae and increasing risk of zoonoses due to human interaction with wildlife and their vectors, surveillance for new *Bartonella* species, especially in new mammalian groups and geographic regions, is important to capture the substantial diversity of this genus and to study the natural cycles of transmission in the hosts.

*Bartonella* spp. infections have now been found in over 60 bat species representing over 40 genera, 11 families, and both suborders from Central and South America, Africa, Europe, and Southeast Asia. Diversification of *Bartonella* species in bats appears to have followed the diversification of bats, with clades of *Bartonella* spp. confined to particular bat families, superfamilies, and suborders [15,16]. Very recent studies of *Bartonella* spp. in bats from Algeria [17], Madagascar and the Union of Comoros [18], French Guiana [19], Saint Kitts [20], South Africa [21], the Republic of Georgia [22], China [23], France and Spain [24], the United States [25], Argentina [26], and Brazil [27] have only added to this substantial diversity. Despite apparent phylogenetic patterns linking bats and bat-associated bartonellae, there is evidence that spillover of bartonellae from bats into other mammals is possible, particularly to humans and dogs [22,28–32].

In the present study, we characterize the diversity of *Bartonella* spp. found in bats sampled in Thailand. Thailand possesses a high diversity of mammal species, particularly bats, many of which may carry zoonotic infections [33]. Bats and their ectoparasites within the region (e.g., China, Laos, Vietnam, Taiwan, and Malaysia) have been laboratory-confirmed to harbor bartonellae [23,32,34,35]. We hypothesized that *Bartonella* species identified in Thailand may likely have phylogenetic relationships with *Bartonella* species previously identified in related bat hosts. We utilized gene sequencing of the citrate synthase gene (*gltA*) and multi-locus sequence typing (MLST) of four additional loci to characterize *Bartonella* spp. isolates from five bat species from four regions in Thailand. MLST is an approach frequently applied to distinguish among *Bartonella* species and epidemiologically relevant strains [36–38]. Utilizing multiple loci can elucidate distant evolutionary relationships, clonal stability, and recombination events among *Bartonella* species [38,39]. This study expands our knowledge of the host range and diversity of *Bartonella* spp. in bats from Southeast Asia and enriches our understanding of the evolutionary history of this diverse genus globally.

## Materials and methods

### *Bartonella* spp. cultures

The current study is based on comparative characterization of 42 cultures of *Bartonella* species (Table 1) selected from isolates obtained from whole blood of bats collected in four Thai provinces: Chiang Rai (north), Kamphaeng Phet (west), Khon Kaen (northeast), and Sa Kaeo (east), as described [40]. Captured bats were anesthetized by intramuscular injection of ketamine hydrochloride (0.05–0.1 mg/g body weight) and euthanized under sedation in accordance with the field protocol approved by the CDC Institutional Animal Care and Use Committee; the CDC IACUC also specifically approved this study. The Supplementary Material (S1 Text) contains additional details regarding sampling locations (Fig A in S1 Text), bat capture, species distributions (Fig B in S1 Text), culturing procedures, and infection prevalence among species and locations (Table A in S1 Text). *Bartonella* spp. bacteria were cultured from blood of 34 (36.6%) of 93 bats distributed among all four provinces and representing five bat species: the wrinkle-lipped free-tailed bat (*Chaerephon plicatus*, Molossidae), the great roundleaf bat (*Hipposideros armiger*, Hipposideridae), the fulvus roundleaf bat (*H*. *fulvus*), the intermediate roundleaf bat (*H*. *larvatus*), and the black-bearded tomb bat (*Taphozous melanopogon*, Emballonuridae).

**Table 1:** Allelic profiles and sequence types (ST) of 30 *Bartonella* isolates from Thai bats.

Host	Province	Isolate	*ftsZ*	*gltA*	ITS	*nuoG*	*rpoB*	ST	Genogroup
*C*. *plicatus*	Sa Kaeo	SK128	1	1	1	1	1	ST1	Cp1
*C*. *plicatus*	Sa Kaeo	SK130	1	1	1	1	1	ST1	Cp1
*C*. *plicatus*	Sa Kaeo	SK144	1	1	1	1	1	ST1	Cp1
*C*. *plicatus*	Sa Kaeo	SK157	1	1	1	1	1	ST1	Cp1
*C*. *plicatus*	Sa Kaeo	SK166	1	1	1	1	1	ST1	Cp1
*C*. *plicatus*	Sa Kaeo	SK168	1	1	1	1	1	ST1	Cp1
*C*. *plicatus*	Sa Kaeo	SK189	1	1	1	1	1	ST1	Cp1
*C*. *plicatus*	Sa Kaeo	SK191	1	1	1	1	1	ST1	Cp1
*C*. *plicatus*	Sa Kaeo	SK202	1	1	1	1	1	ST1	Cp1
*C*. *plicatus*	Sa Kaeo	SK170	1	2	1	1	1	ST2	Cp1
*C*. *plicatus*	Sa Kaeo	SK194	2	3	2	2	2	ST3	Cp2
*C*. *plicatus*	Sa Kaeo	SK197	2	3	3	2	2	ST4	Cp2
*C*. *plicatus*	Sa Kaeo	SK163	3	4	4	3	3	ST5	Cp3
*C*. *plicatus*	Sa Kaeo	SK165	3	4	5	3	3	ST6	Cp3
*C*. *plicatus*	Sa Kaeo	SK180	3	4	4	3	3	ST5	Cp3
*C*. *plicatus*	Sa Kaeo	SK198a	3	4	4	3	3	ST5	Cp3
*H*. *armiger*	Khon Kaen	KK182	4	5	6	5	4	ST7	H3
*H*. *larvatus*	Kamphaeng Phet	KP270	4	6	6	4	4	ST8	H3
*H*. *larvatus*	Kamphaeng Phet	KP215	5	5	7	4	5	ST9	H3
*H*. *fulvus*	Chiang Rai	CR224	6	7	8	6	6	ST10	H3
*H*. *larvatus*	Kamphaeng Phet	KP277	7	8	9	7	7	ST11	H2
*Hipposideros* sp.	Sa Kaeo	KP174	8	9	10	4	8	ST12	H1
*H*. *larvatus*	Kamphaeng Phet	KP287a	9	9	10	8	7	ST13	H1
*H*. *larvatus*	Kamphaeng Phet	KP216a	8	9	10	8	8	ST14	H1
*H*. *larvatus*	Kamphaeng Phet	KP268a	8	9	10	8	8	ST14	H1
*H*. *larvatus*	Kamphaeng Phet	KP292	8	9	10	8	8	ST14	H1
*H*. *armiger*	Khon Kaen	KK200a	8	9	10	8	9	ST15	H1
*H*. *larvatus*	Kamphaeng Phet	KK290	8	9	10	8	9	ST15	H1
*H*. *larvatus*	Kamphaeng Phet	KP293a	9	9	10	8	8	ST16	H1
*T*. *melanopogon*	Kamphaeng Phet	KP283b	10	10	11	9	10	ST17	Tm

Genogroups were determined using a combination of sequence typing, phylogenetic analysis, and population clustering.

### DNA purification and multi-locus sequence typing (MLST)

A suspension of pure cultured isolate was heated to 95°C for 10 min followed by 1 min centrifugation at 8000 rpm for the lysed cells to precipitate. The supernatant was then moved to a clean microcentrifuge tube for storage until examination. Isolates were initially verified as *Bartonella* spp. and genotyped by PCR amplification of a fragment of the citrate synthase gene (*gltA*) following previous studies [41,42]. PCR products were separated by 1.5% gel electrophoresis and visualized by ethidium bromide staining Positive PCR products were purified using the QIAquick PCR Purification Kit (QIAGEN, Valencia, CA) according to manufacturer protocols and sequenced in both directions with the same primers on an Applied Biosystems Model 3130 Genetic Analyzer (Applied Biosystems, Foster City, CA). Obtained forward and reverse reads were assembled using Lasergene v11 (DNASTAR, Madison, WI). Sequences were first verified as *Bartonella* spp. DNA using BLAST (National Center for Biotechnology Information, Bethesda, MD) and closely matching sequences (>95% sequence identity were downloaded as references.

Further characterization of 30 isolates from *Hipposideros* spp., *C*. *plicatus*, and *T*. *melanopogon* was performed using MLST of four additional loci (*ftsZ*, *nuoG*, *rpoB*, and ITS) previously used for characterization of *Bartonella* strains [38,39,41,43–46]. Primers and associated references for protocols are provided in Table 2. PCR purification, sequencing, and alignment used the same methods as with *gltA*. All sequences were aligned with MAFFT v7.187 using the local, accurate L-INS-I algorithm [47], trimmed to equal lengths with Gblocks v0.91b [48], and compared with other *Bartonella* strains from bats, bat ectoparasites, and known *Bartonella* species (Table B in S1 Text).

**Table 2:** Oligonucleotide primers used for MLST characterization of *Bartonella* strains.

Locus	Product	Primer	Primer sequence (5’ to 3’)
*ftsZ*	Cell division protein	Bfp1 (f) [43]	ATTAATCTGCAYCGGCCAGA
		Bfp2 (r) [43]	ACVGADACACGAATAACACC
*gltA*	Citrate synthase	BhCS781.p (f) [41]	GGGGACCAGCTCATGGTGG
		BhCS1137.n (r) [41]	AATGCAAAAAGAACAGTAAACA
*nuoG*	NADH dehydrogenase *γ*-subunit	nuoG1F (f) [44]	GGCGTGATTGTTCTCGTTA
		nuoG1R (r) [44]	CACGACCACGGCTATCAAT
*rpoB*	RNA polymerase *β*-subunit	1400F (f) [45]	CGCATTGGCTTACTTCGTATG
		2300R (r) [45]	GTAGACTGATTAGAACGCTG
ITS	16S–23S internal transcribed spacer	325s (f) [46]	CTTCAGATGATGATCCCAAGCCTTCTGGCG
		1100as (r) [46]	GAACCGACGACCCCCTGCTTGCAAAGA

Listed references include detailed thermocycler protocols. For each primer set, (f) indicates the forward primer and (r) indicates the reverse primer.

Each unique variant was assigned to a sequence type (ST) based on the allelic profile (Table 1). Nucleotide polymorphisms and diversity of the five MLST loci were examined in MEGA v7.0.21 [49] among all 17 STs. The correct open reading frame for protein coding loci (*ftsZ*, *gltA*, *nuoG*, and *rpoB*) was determined by checking all starting positions and choosing the frame that contained no premature stop codons. Ratios of non-synonymous to synonymous substutitions (dN/dS) were calculated using the Nei-Gojobori method for each of the loci to check for evidence of selection. Nucleotide diversity (π) and mean pairwise sequence distance were estimated for the five loci separately and for concatenated sequences; sequence distances were calculated as the number of substitutions per site. ITS sequences contained gap regions, so the total lengths of sequences for this locus and concatenated sequences include gaps.

### Phylogenetic analysis

The optimal substition model was selected with jModelTest v2.1.6 [50] using Akaike’s information criterion corrected for finite sample sizes (AICc) [51]. The generalized time-reversible model with four gamma-distributed rate categories and a proportion of invariant sites (GTR+Γ+I) was the best available model for all loci. Separate maximum likelihood (ML) phylogenetic trees for each locus (*gltA*, *ftsZ*, *nuoG*, *rpoB*, and ITS) were created for the 30 isolates characterized by multiple loci to visualize clustering of strains and to identify potential recombination events among loci. Trees were generated without reference sequences with RAxML v8.2.10 [52] using 1000 bootstrap replicates to estimate node support.

A Bayesian phylogeny of *gltA* sequences from Thai bats, known *Bartonella* species, and select *Bartonella* strains from bats and their ectoparasites from the Americas [19,25,34,53], Europe [22,31,54,55], Africa [18,38,42], and Southeast Asia [23,32,35]. This selection of bat-associated *Bartonella* strains is representative of phylogenetic clades found in previous phylogenies of *gltA* sequences from bats [16,22]. Closely matching sequences (>95% sequence identity) based on the initial BLAST search from free-ranging dogs from Thailand [28] and other bats and ectoparasites from Vietnam, Laos, and Malaysia [34,35] were included in the phylogenetic analysis. The *gltA* phylogeny was inferred by Markov chain Monte Carlo (MCMC) sampling in BEAST v1.8.4 [56,57] using the GTR+Γ+I model. Well-supported clades (posterior probability > 0.9) of bat-associated *Bartonella* strains were collapsed within the *gltA* phylogeny and labeled with the host families and countries represented in the clade to reduce complexity.

A second Bayesian tree was generated from concatenated sequences of five loci (*ftsZ*, *gltA*, *nuoG*, *rpoB*, and ITS) from Thai bats (30 isolates), known *Bartonella* species, and other *Bartonella* strains from bats [31,32,38,42]. The selection of bat strains for the multi-locus phylogeny was taken from the same set of strains used in the *gltA* analysis, with the added restriction that the strains had been characterized by at least two of the loci we had sequenced from Thai bats. This restricted set contained strains from the Americas [19,25], Europe [22,31,55], Africa [38,42], and Southeast Asia [32] and is representative of clades found in previous multi-locus phylogenies of bat-associated *Bartonella* species [22,38]. For the multi-locus phylogeny, we used separate partitions for each of the five loci; each locus was analyzed under the GTR+Γ+I substitution model, but parameters were allowed to vary for each partitioned locus. All loci were linked with the same clock model and speciation model. GenBank accession numbers for all sequences used in the *gltA* and multi-locus phylogenetic analyses are listed in the Supplementary Material (Table B in S1 Text).

For both BEAST phylogenies, we set the number of MCMC iterations to 2×10^8^, sampling every 2×10^4^th iteration. No codon partitions were used for either the *gltA* or multi-locus analyses due to the short sequence length of all loci which could substantially reduce the effective sample size of estimated parameters for each codon position. A strict molecular clock was chosen for both phylogenies because we did not seek to accurately estimate branch times. Additionally, all of the isolates from Thai bats were cultured around the same date and therefore could not be used to calibrate another clock model. We chose to use the birth-death model with incomplete sampling to represent patterns of speciation in the phylogeny [58]. All priors were kept at the default, diffuse settings for both the *gltA* and multi-locus analyses (see S1 Text for details). Three separate chains were run and effective sample sizes (ESS) and mixing of parameters during MCMC sampling were assessed using Tracer v1.6 [56]. Chains were then combined and the maximum clade credibility tree was found using TreeAnnotator [56,57].

### Recombination and admixture analysis

To assess the level of recombination among sequence types, a phylogenetic network was inferred using the Neighbor-Net algorithm in SplitsTree v4.13.1 [59] from concatenated sequences of all five loci (*ftsZ*, *gltA*, *nuoG*, *rpoB*, and ITS) from the 30 *Bartonella* isolates from Thai bats evaluated by MLST. The pairwise homoplasy index [60] was calculated in SplitsTree to test for significant recombination among the sequence types. Bayesian population clustering was performed with STRUCTURE v2.3.4 [61] using concatenated sequences of all five loci from the 30 isolates evaluated by MLST. The program was run five times for each value of K (the number of population clusters) ranging from 3–10 for 5×10^4^ iterations and 5×10^4^ burn-in iterations using the admixture model with correlated allele frequencies. Convergence of MCMC chains for each run was assessed by visual analysis of trace diagrams for all measured parameters. The optimal value of K was estimated according to the ΔK method [62] with STRUCTURE HARVESTER v0.6.94 [63]. We did not evaluate K = 2 due to our prior observation of at least three distinct clades in the multi-locus phylogeny based on host genus (*Hipposideros* spp., *Chaerephon* sp., and *Taphozous* sp.) and a recently observed bias towards the selection of K = 2 as the optimal number of populations in studies that use the ΔK method [64].

### Nucleotide sequence accession numbers

Unique alleles from this study were submitted to GenBank with the following accession numbers: KY232154 to KY232182 and MF288092 (*ftsZ*), KY232183 to KY232224 (*gltA*), KY232254 to KY232282 and MF288099 (*nuoG*), KY232283 to KY232311 and MF288103 (*rpoB*), and KY232225 to KY232253 and MF288133 (ITS).

## Results

### Analysis of *gltA* genotypes

**Fig 1:**
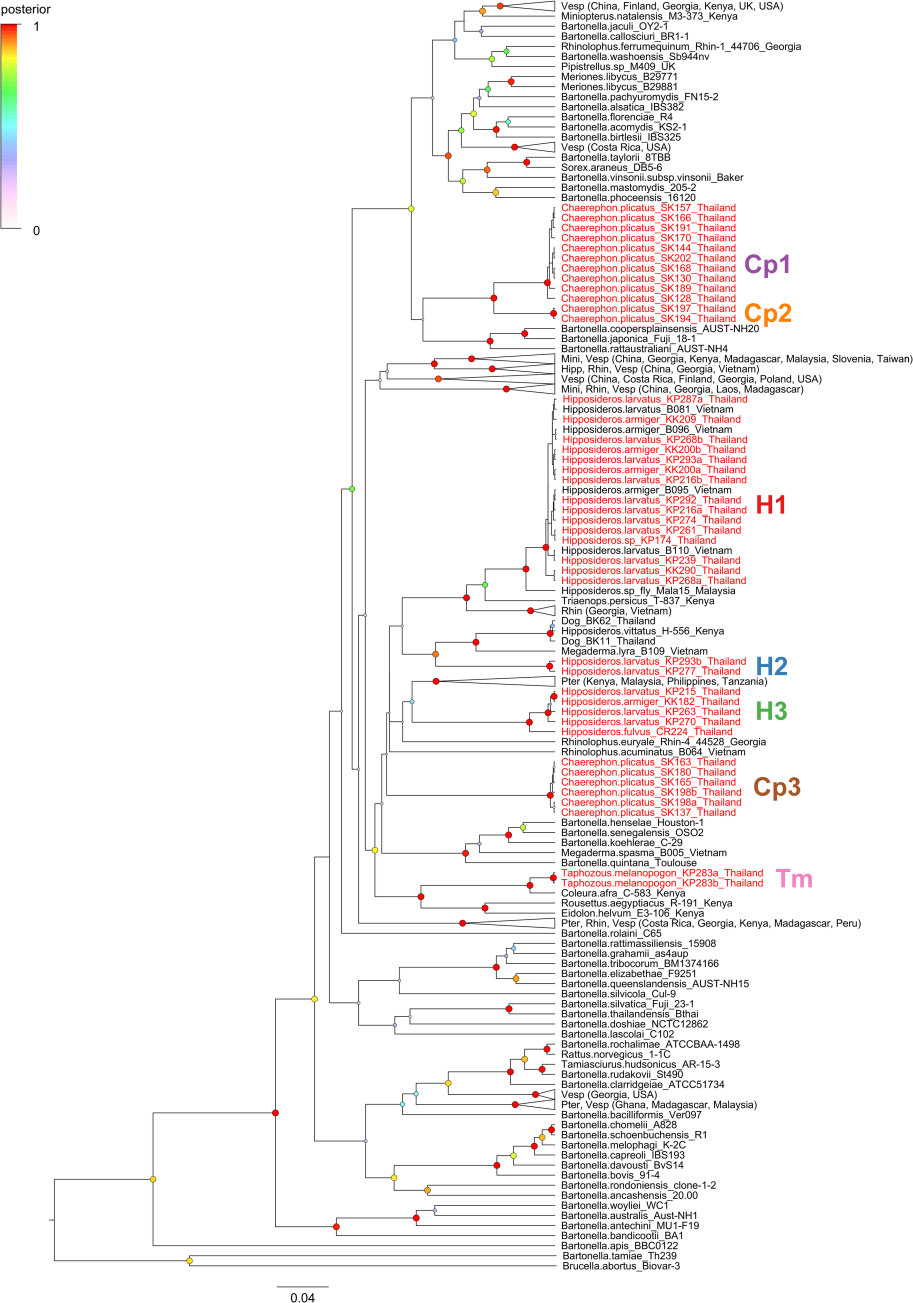
Phylogenetic relationships among citrate synthase (*gltA*) sequences of *Bartonella* strains from Thai bats. The phylogeny was inferred by Bayesian analysis in BEAST using the GTR+Γ+I substitution model and a birth-death speciation model with incomplete sampling. Branch lengths are in substitutions per site and posterior probabilities of nodes are indicated by the size and color of circles at each node. Tip labels for *Bartonella* strains from Thai bats are colored red and distinct genogroups are marked to the right of the tree. Well-supported clades (posterior probability >0.9) of bat-associated *Bartonella* strains were collapsed and labeled with the bat families and countries represented in the clade. Bat family abbreviations: Hipp, Hipposideridae; Mini, Miniopteridae; Pter, Pteropodidae; Rhin, Rhinolophidae; Vesp, Vespertilionidae. Other *Bartonella* strains from bats distinct from these collapsed clades are marked with the host species and country of origin.

Initial phylogenetic analysis based on *gltA* sequences (Fig 1) demonstrated the presence of three genogroups found in *Hipposideros* spp. bats (H1-3), three other genogroups found in *Chaerephon plicatus* (Cp1-3), and a distinct genogroup in *Taphozous melanopogon* (Tm). Posteriors distributions for the *gltA* tree likelihood and all estimated parameters of the substitution model and the birth-death speciation model converged and had sufficient effective sample sizes (ESS > 200) for each of the three chains separately and combined. Sequences from one genogroup (H1) from Thai *H*. *armiger* and *H*. *larvatus* were nearly identical (>99% sequence identity) to sequences found in *H*. *armiger* and *H*. *larvatus* from Vietnam [35]. This genogroup formed a well-supported clade (posterior probability = 1) with other sequences from hipposiderid and rhinolophid bats and their bat flies from Vietnam [35], Malaysia [34], Kenya [42], and Georgia [22]. Another group of sequences from *H*. *larvatus* (H2) formed a clade (posterior probability = 0.93) with *Bartonella* genotypes from *Megaderma lyra* in Vietnam [35], *Hipposideros vittatus* (previously identified as *H*. *commersoni*) from Kenya [42,65], and community dogs from Thailand [28], with sequence identities ranging from 88–90% in this clade. The third genogroup (H3) from *H*. *armiger*, *H*. *fulvus*, and *H*. *larvatus* clustered (88–90% sequence identity) with *Bartonella* species found in pteropodid bats: *Bartonella* species found in *Eidolon helvum* from Africa [38,42], sequences from bat flies collected from *Pteropus hypomelanus* in Malaysia [34], and bat flies from *Ptenochirus jagori* and *Harpyionycteris whiteheadi* in the Philippines [34]. However, the posterior support for this clade was only 0.45 based on data from *gltA* sequences alone.

Two genogroups found in *C*. *plicatus* (Cp1 and Cp2) are closely related (94% sequence identity) to each other and formed a well-supported clade (posterior probability = 1). These two genogroups were more distantly related (87% sequence identity) to the third cluster (Cp3). Finally, the single genogroup from *T*. *melanopogon* (Tm) is very closely related (95% sequence identity) to the *Bartonella* strain found in *Coleura afra*, another emballonurid bat, in Kenya [42]. These two groups form a well-supported clade (posterior probability = 1) with *Bartonella* species from African pteropodid bats (*Eidolon helvum* and *Rousettus aegyptiacus*) [42]. Sequence divergence was ≤3.1% within a genogroup and 6.2–16.2% among genogroups. All of these separate clusters are sufficiently distinct from one another based on *gltA* sequences (<96% DNA similarity) to be considered putative new *Bartonella* species [66]. However, as we acknowledged above, genogroup H1 appears to have been discovered previously in *H*. *armiger* and *H*. *larvatus* from Vietnam [35], but was not cultured or characterized by additional genetic loci.

### Allelic profiles and sequence types

Based on allelic profiling, the MLST analysis distinguished 17 sequence types (ST) among the 30 isolates (Table 1). All five sequenced loci distinguished either eight or nine alleles among the isolates. Genogroups Cp1–3 contained isolates from only *C*. *plicatus*. Genogroup Cp1 was almost entirely clonal with nine isolates characterized as ST1 and a single isolate of ST2; the distance among STs based on concatenated sequences of the five loci was 0.036%. Genogroup Cp2 had two distinct isolates, characterized as ST3 and ST4, with a distance of 0.035% among STs. Genogroup Cp3 was also nearly clonal, with three isolates characterized as ST5 and one isolate as ST6; the distance among STs was 0.14%.

Genogroup H1 was comprised of isolates from *H*. *armiger*, *H*. *larvatus*, and another *Hipposideros* sp. bat. This group was the most variable, with five distinct STs (ST12–16) with a maximum sequence distance of 3.9%. Genogroup H2 from *H*. *larvatus* was a single, distinct type (ST11). Genogroup H3 had isolates from *H*. *armiger*, *H*. *fulvus*, and *H*. *larvatus* with four distinct sequence types (ST7-10) with a maximum sequence distance of 2.0%.

Some of the unique sequence types arose from apparent homologous recombination events among genogroups, highlighted in the individual gene trees (Figs C-G in S1 Text). One strain from *H*. *larvatus* (isolate KP287a, ST13) clustered with genogroup H1 for all loci except for *rpoB* where it clustered with genogroup H2. Another strain from the *Hipposideros* sp. bat (isolate KP174, ST12) clustered with genogroup H1 for all loci except *nuoG* where it clustered with genogroup H3. Even with these recombinant strains, genogroups remained distinct, with ≤3.9% sequence distance within a genogroup and 6.4–15.8% distance among genogroups.

### Patterns of selection and diversity in nucleotide sequences

The five analyzed loci revealed different levels of variation over the length of sequenced fragments (Table 3), ranging from 21.6% variable sites for *ftsZ* to 45.3% for ITS. Mean pairwise sequence distances ranged from 8.3% for *ftsZ* to 22.8% for ITS. Nucleotide diversity showed a similar pattern, with values ranging from 8.0% for *ftsZ* to 12.7% for ITS. Based on concatenated sequences from all five loci, there were 895 (28.7%) variable sites among the 30 STs over the length of the alignment with 9.5% nucleotide diversity and a mean pairwise distance of 11.0%. Calculated dN/dS ratios from protein coding loci were generally low, ranging from 0.03 for *ftsZ* to 0.09 for *gltA*, indicating that purifying selection is dominant for these genes.

**Table 3:** Nucleotide polymorphism and diversity among *Bartonella* strains from Thai bats.

Genes	Size (bp)	# alleles	V	N	V (%)	N (%)	dN	dS	dN/dS	π (%)	Mean pairwise distance (%)
*ftsZ*	886	10	191	17	21.6	5.8	0.01	0.31	0.03	8.0	8.3
*gltA*	356	10	101	19	28.4	16.1	0.03	0.32	0.09	9.3	9.3
*nuoG*	353	9	86	10	24.4	8.5	0.02	0.31	0.06	8.6	8.9
*rpoB*	833	10	205	20	24.6	7.2	0.02	0.36	0.04	9.0	9.9
ITS	689	11	312	NA	45.3	NA	NA	NA	NA	12.7	22.8
Concatenate, no ITS	2428	15	583	66	24.0	8.2	0.02	0.33	0.05	8.6	9.1
Concatenate	3117	17	895	NA	28.7	NA	NA	NA	NA	9.5	11.0

Values are calculated from all individuals (n = 30). ITS is not a protein coding locus and contains large insertions and deletions, thus only nucleotide diversity is calculated. The length of ITS sequences is based on aligned sequences, which includes gaps. π, average number of nucleotide differences per site; V, number of variable sites; N, number of non-synonymous sites; S, number of synonymous sites; dS, number of synonymous changes per synonymous site; dN, number of non-synonymous changes per non-synonymous site; NA, not applicable. Mean pairwise distance was calculated using the number of substitutions per site and dN and dS were calculated using the Nei-Gojobori method.

### Phylogenetic analysis of multiple loci

**Fig 2:**
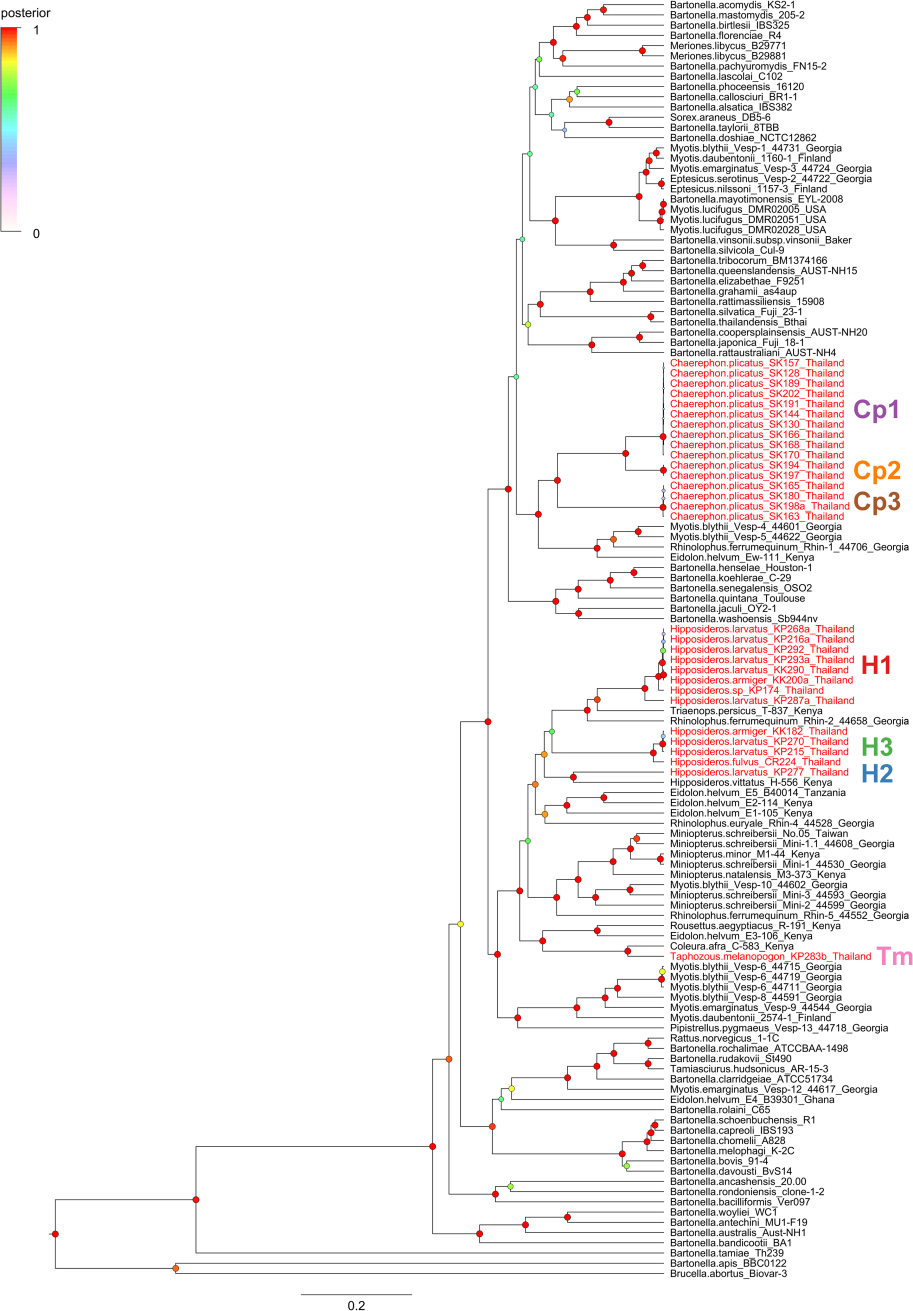
Phylogenetic relationships among *Bartonella* genogroups from Thai bats, other strains from bats, and named *Bartonella* species assessed by multiple loci. The Bayesian tree was inferred in BEAST by partitioned analysis of five loci (*ftsZ*, *gltA*, *nuoG*, *rpoB*, and ITS) using unlinked GTR+Γ+I substitution models for each locus and a linked birth-death speciation model with incomplete sampling. Sequences were assembled from named *Bartonella* species, *Bartonella* strains from bats (those characterized by at least two loci), and 30 isolates from Thai bats. Branch lengths are in substitutions per site and posterior probabilities of nodes are indicated by the size and color of circles at each node. Tip labels for *Bartonella* strains from Thai bats are colored red and distinct genogroups are marked to the right of the tree. *Bartonella* strains from bats are labeled with the host species and country of origin.

The Bayesian tree assembled by partitioned analysis of *ftsZ*, *gltA*, *nuoG*, and *rpoB* sequences (Fig 2) clarified the phylogenetic position of the seven genogroups identified by *gltA* sequences relative to other *Bartonella* strains associated with bats. As with the *gltA* analysis, the posterior distributions for all relevant model parameters (for each partitioned locus) and the combined tree likelihood converged and had large effective sample sizes (ESS > 200). Posterior support for nodes was higher across the tree as compared to the *gltA* due to the added sequence information. The multi-locus phylogeny shows that genogroups Cp1-3 form a unified clade (posterior probability = 0.99) that is part of a larger clade (posterior probability = 1) with a *Bartonella* species isolated from *Eidolon helvum* in Africa [38,42] and multiple species isolated from *Myotis blythii* and *Rhinolophus ferrumequinum* in Georgia [22].

Genogroup H1 formed a clade (posterior probability = 1) with *Bartonella* species from *Triaenops persicus* in Kenya [42] and *Rhinolophus ferrumequinum* in Georgia [22] while genogroup H2 was linked (posterior probability = 1) to the *Bartonella* species from *Hipposideros vittatus* from Kenya [42]. All three genogroups from *Hipposideros* spp. bats (H1-3) were closely linked (posterior probability = 0.91) and more distantly related to *Bartonella* species from *Eidolon helvum* in Africa and *Rhinolophus euryale* in Georgia [22,38,42]. Strains KP287a from *H*. *larvatus* and KP174 from a *Hipposideros* sp. bat diverge slightly from the rest of genogroup H1 due to recombination events with genogroups H2 and H3, respectively. Similar to the *gltA* phylogeny, genogroup Tm was very similar to the *Bartonella* species from *Coleura afra* in Kenya and more distantly related to *Bartonella* species from other African fruit bats, *Eidolon helvum* and *Rousettus aegyptiacus* [42]. Genogroups H1-3 and Tm are all members of a large and well-supported clade (posterior probability = 1) composed entirely of bat-associated *Bartonella* species from Africa and Eurasia recognized in previous multi-locus phylogenetic analyses [22,38].

### Recombination and admixture analyses

**Fig 3:**
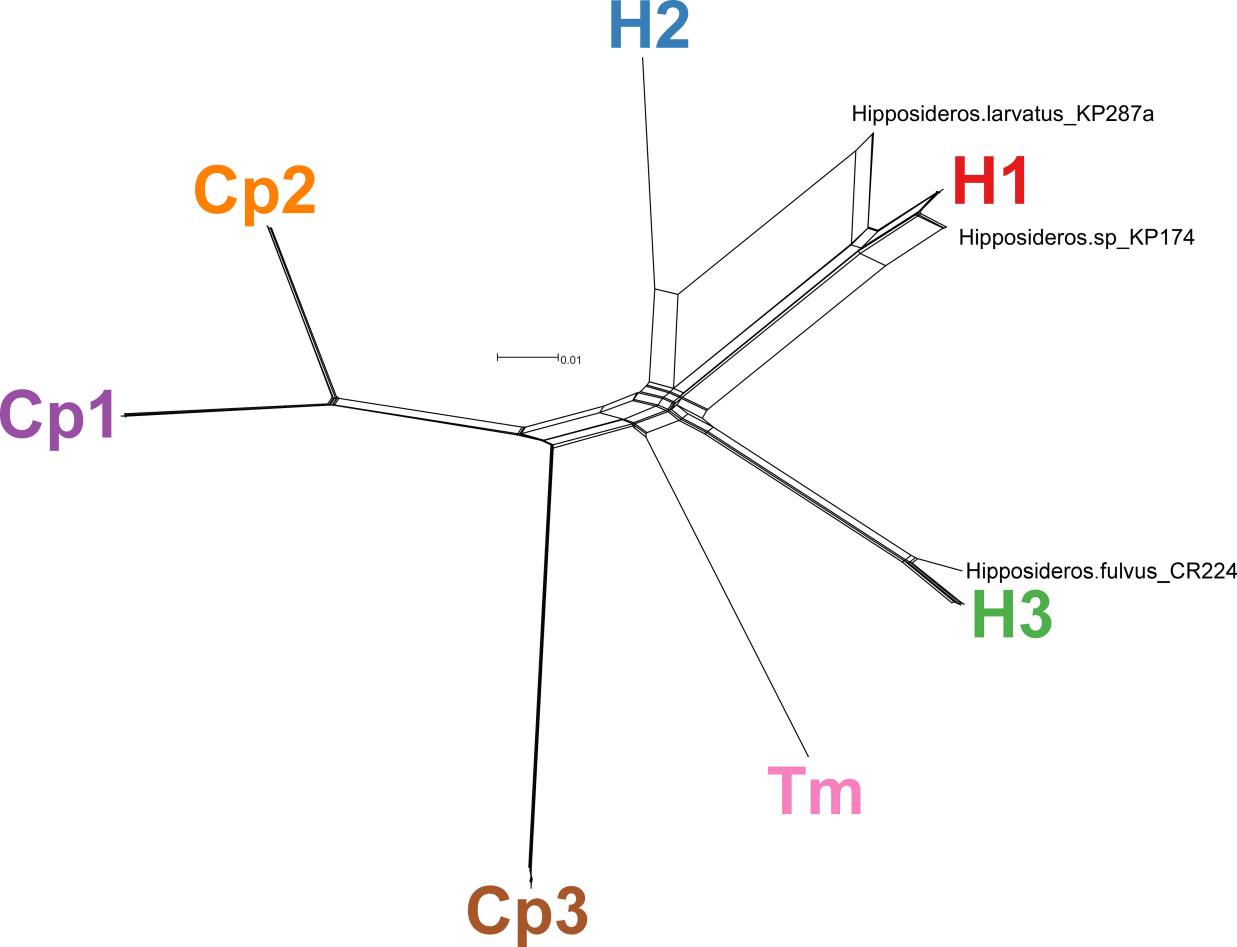
Network phylogeny of *Bartonella* strains from Thai bats. The network was inferred using the NeighborNet algorithm in SplitsTree based on concatenated sequences of five loci (*ftsZ*, *gltA*, *nuoG*, *rpoB*, and ITS) from 30 *Bartonella* isolates analyzed by MLST. Distinct genogroups are named next to clusters of isolates. Recombinant isolates are labeled individually.

**Fig 4:**
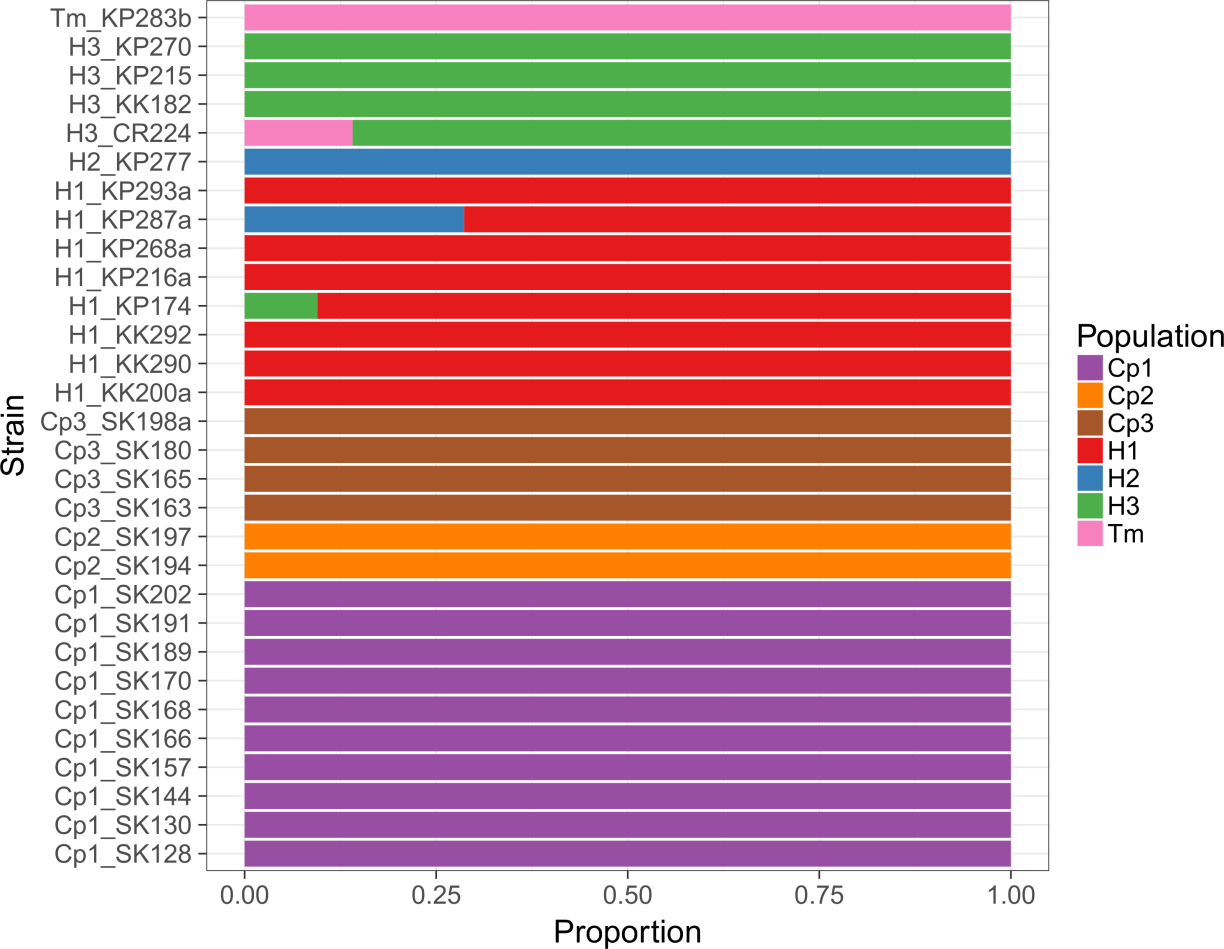
Inferred admixture events among *Bartonella* strains in Thai bats. Estimates of allele proportions from STRUCTURE based on the optimal clustering of K = 7 using concatenated sequences of five loci (*ftsZ*, *gltA*, *nuoG*, *rpoB*, and ITS) from 30 *Bartonella* isolates analyzed by MLST. Populations are named according to genogroups identified by phylogenetic analyses.

The network phylogeny from SplitsTree (Fig 3) generated from concatenated sequences of five loci (*ftsZ*, *gltA*, *nuoG*, *rpoB*, and ITS) supported the distinction between the seven genogroups (Cp1-3, H1-3, and Tm). However, the pairwise homoplasy index [60] test found significant recombination among the isolates (mean = 0.19, variance = 6.6×10^−6^, p-value < 0.0001). These recombination events can been seen in the web-like linkage between genogroups H1 and H2 for strain KP287a and the linkage between genogroups H1 and H3 for strain KP174. The optimal number of populations within the isolates was seven according to the ΔK method [62,63] after Bayesian clustering analysis using STRUCTURE [61]. All seven of these populations matched with the genogroups distinguished by the MLST profiles and phylogenetic analysis. The clustering analysis showed that strain KP287a was mostly composed of genogroup H1 with some genetic material from genogroup H2 and strain KP174 was almost entirely composed of genogroup H1 with some admixture with genogroup H3. This admixture is confirmed by the maximum likelihood analysis of the five sequenced loci (Figs C-G in S1 Text), showing that the *nuoG* sequence of strain KP174 clustered with genogroup H3 and the *rpoB* sequence of strain KP287a clustered with genogroup H2. The relative amount of admixture (Fig 4) in these recombinant strains was also proportional to the size of *nuoG* (353 bp) and *rpoB* (833 bp) sequences. STRUCTURE analysis was also able to discern some admixture between strain CR224 from *H*. *fulvus* in Chiang Mai (genogroup H3) with genogroup Tm. This admixture was not as obvious as with strains KP287a and KP174, but is observable from the distinction of strain CR224 from all other members of genogroup H3 at the five sequenced loci (Figs C-G in S1 Text) and some web-like connections between genogroups H3 and Tm in the network phylogeny (Fig 3).

## Discussion

*Bartonella* is a highly diverse genus of bacteria and bats have been distinguished as particularly reliable sources of novel *Bartonella* species. The present study focused on characterization of *Bartonella* isolates from bat species in Thailand. We identified seven novel *Bartonella* genogroups in five species of bats using sequences of the *gltA* gene. Genogroups H1 through H3 were found in roundleaf bats (*H*. *armiger*, *H*. *larvatus*, and *H*. *fulvus*). Genogroups Cp1 through Cp3 were found in free-tailed bats (*C*. *plicatus*) and genogroup Tm was found in sheath-tailed bats (*T*. *melanopogon*).

Comparison with previous *gltA* sequences on GenBank showed that genogroup H1 had been previously detected in *H*. *armiger* and *H*. *larvatus* in Vietnam [35] and is closely related to sequences found in a bat fly (*Phthiridium fraterna*) removed from a *Hipposideros* sp. bat from Malaysia [34] and sequences found in *Rhinolophus* spp. from Vietnam and Georgia [22,35]. Genogroup H2 was found to be related to sequences found in *Hipposideros vittatus* (previously reported as *H*. *commersoni*) from Kenya [42,65], *Megaderma lyra* from Vietnam [35], and community dogs from Thailand [28]. Genogroup H3 clustered with *Bartonella* species identified in *Eidolon helvum* in Africa [38,42] and bat flies from *Pteropus hypomelanus*, *Ptenochirus jagori*, and *Harpyionycteris whiteheadi* in Malaysia and the Philippines [34]. Genogroup Tm was found to be very closely related to a *Bartonella* species from *Coleura afra* in Kenya [42].

Phylogenetic analysis of multiple loci confirmed that genogroups H1-3, Cp1-3, and Tm are divergent enough to be considered separate *Bartonella* species according to previously established criteria based on individual loci, with genogroups differing by 6.5–15.6% sequence identity [66]. Most genogroups displayed clonal behavior with very little variation at multiple loci, however genogroups H1 and H3 showed measurable genetic variation at several loci. Additionally, these groups showed some evidence of homologous recombination. These heterogeneous patterns of genetic variation and homologous recombination have been observed in other *Bartonella* species found in bats [38].

Host specificity of *Bartonella* species in bats has been a subject of some discussion [42,67,68]. As more studies have been performed, it is clear that *Bartonella* species are typically shared among bats in the same families, superfamilies, and suborders [16]. Transmission may be facilitated by shared ectoparasites when species roost in sympatry [22]. All five of the focal bat species in this study inhabit caves and manmade structures and host a variety of ectoparasite families. *Chaerephon plicatus* has been found infested with bat flies (Diptera: Nycteribiidae), fleas (Siphonaptera: Ischnopsyllidae), ticks (Ixodida: Argasidae, Ixodidae), mites (Trombidiformes: Myobiidae; Sarcoptiformes: Sarcoptidae, Listrophoridae), and bat bugs (Hemiptera: Cimicidae) in Malaysia, the Philippines, and Thailand [69–72]. *Taphozous melanopogon* hosts bat flies (Diptera: Nycteribiidae, Streblidae), ticks (Ixodida: Argasidae), mites (Trombidiformes: Myobiidae, Trombiculidae; Sarcoptiformes: Listrophoridae; Mesostigmata: Macronyssidae, Spinturnicidae), and bat bugs (Hemiptera: Polyctenidae) in Thailand, Malaysia, Burma, Sri Lanka, Indonesia, and India [69,73–77]. Bat flies (Diptera: Nycteribiidae, Streblidae), ticks (Ixodida: Argasidae, Ixodidae), and mites (Trombidiformes: Myobiidae, Trombiculidae; Sarcoptiformes: Listrophoridae; Mesostigmata: Macronyssidae, Spinturnicidae) are known to parasitize *Hipposideros* spp., including *H*. *armiger* and *H*. *larvatus* in Vietnam, Indonesia, China, Thailand, Malaysia, and Burma [69,73,78–87]. Of these ectoparasite groups, nycteribiid and streblid bat flies, cimicid bugs, ischnopsyllid fleas, argasid and ixodid ticks, and macronyssid and spinturnicid mites are suspected vectors of *Bartonella* spp. in bats [17,34,53,88–93]. Trombiculid mites parasitizing rodents have been found harboring *Bartonella* spp. and may also be vectors of *Bartonella* spp. in bats [94,95].

Based on our observations of separate *Bartonella* species infecting *Chaerophon plicatus*, *Taphozous melanopogon*, and *Hipposideros* spp., we may surmise that transmission among these genera is uncommon, due in part to the specificity of *Bartonella* spp. to related host species and perhaps reinforced by the specificity of ectoparasites to their bat hosts. Although our focal species share a range of ectoparasite families, there are likely specific associations of ectoparasites to one or a few related bats. There are few data available concerning the host range of ectoparasites in Southeast Asia, so more study is warranted to fully understand the ecology and transmission dynamics of *Bartonella* spp. in bats and their ectoparasites in this region.

## Supporting information

S1 Text Supplementary material. This file includes additional information on field and laboratory methods and supplementary tables and figures.
(HTML)
