# Supplementary material for: Diversity and phylogenetic relationships among Bartonella strains from Thai bats
Source: PLoS One. 2017 Jul 20;12(7):e0181696. doi: 10.1371/journal.pone.0181696 (PMC5519213; doi:10.1371/journal.pone.0181696)
Supplement: S1 Text — This file includes additional information on field and laboratory methods and supplementary tables and figures. (HTML) [file pone.0181696.s001.html]

Supplementary Material


# Supplementary Material

### *Diversity and phylogenetic relationships among Bartonella strains from Thai bats*

#### *Clifton D. McKee*

Division of Vector-Borne Diseases, Centers for Disease Control and Prevention, Fort Collins, CO 80521, USA; Department of Biology, Colorado State University, Fort Collins, CO 80523, USA  

#### *Michael Y. Kosoy, Ying Bai, Lynn M. Osikowicz*

Division of Vector-Borne Diseases, Centers for Disease Control and Prevention, Fort Collins, CO 80521, USA  

#### *Amy T. Gilbert*

Division of High-Consequence Pathogens and Pathology, Centers for Disease Control and Prevention, Atlanta, GA 30333, USA; National Wildlife Research Center, USDA/APHIS/Wildlife Services, Fort Collins, CO 80521, USA  

#### *Richard Franka*

Division of High-Consequence Pathogens and Pathology, Centers for Disease Control and Prevention, Atlanta, GA 30333, USA  

#### *Sumalee Boonmar*

Faculty Sciences and Public Health, Rajapruk University, Nonthaburi 11130, Thailand  

#### *Charles E. Rupprecht*

LYSSA LLC, Lawrenceville, GA 30044, USA  

#### *Leonard F. Peruski*

Center for Global Health, Centers for Disease Control and Prevention, Atlanta, GA 30333, USA

#### Supplementary Materials and Methods

##### Sampling locations and bat capture

Bats were captured from locations in four provinces (Fig A): Wat Tham Phra Cave in Mueang Chiang Rai District, Chiang Rai Province; Sia Cave in Khlong Lan National Park, Khlong Lan District, Kamphaeng Phet Province; a cave in Phu Pha Man District, Khon Kaen Province; and Khao Chakan Cave, Mueang Sa Kaeo District, Sa Kaeo Province.

Bat capture followed the approach used previously (Kuzmin et al. 2008; M. Y. Kosoy et al. 2010; Bai et al. 2012). Briefly, bat sampling sites in each province were chosen based on available information about bat roosts and observations of bat activity in the area. Bats were collected using hand nets or manually in caves while mist nets were used to capture foraging bats. Captured bats were anesthetized by intramuscular injection of ketamine hydrochloride (0.05-0.1 mg/g body weight) and euthanized under sedation in accordance with the field protocol approved by the CDC Institutional Animal Care and Use Committee; the CDC IACUC also specifically approved this study. Bats were weighed, sexed, and identified phenotypically to species using available field keys; some individuals could only be identified to the genus level. Bats were exsanguinated by cardiac puncture and blood was stored in sterile plastic tubes. Blood samples were transported from the field on dry ice.

**Fig A. Map of bat sampling locations in Thailand.** Bats were sampled in four provinces: Chiang Rai, Kamphaeng Phet, Khon Kaen, and Sa Kaeo. Counts of bat species captured in each location are shown in Figure S2.

##### *Bartonella* spp. culturing

Blood samples from bats were cultured following previously published protocols for *Bartonella* bacteria (M. Y. Kosoy et al. 1997). Briefly, bat blood was diluted 1:4 in brain heart infusion broth with 5% fungizone (amphotericin B) and 100 µl of the sample was placed on agar plates supplemented with 10% rabbit blood. Plates were then incubated at 35 ºC with 5% \(\text{CO}\_{2}\) for up to five weeks, checking periodically for growth. Bacterial colonies that resembling those typical for *Bartonella* were passaged onto new plates to obtain pure cultures. Morphologically unique colonies obtained from the same sample were subpassaged in an attempt to find possible *Bartonella* coinfections. All isolates were collected in 10% glycerol solution. Cultures were then sent to the CDC Division of Vector-Borne Diseases in Fort Collins, Colorado for genetic characterization.

##### Priors used in Bayesian phylogenetic analyses

For all phylogenetic analyses using BEAST v1.8.4 on the five genetic loci analyzed (*ftsZ*, *gltA*, *nuoG*, *rpoB*, and ITS), the following default, diffuse priors were used for the GTR+Γ+I and birth-death models:

- A-C substitutions ~ gamma(0.05, 10), initial value = 1
- A-G substitutions ~ gamma(0.05, 20), initial value = 1
- A-T substitutions ~ gamma(0.05, 10), initial value = 1
- C-G substitutions ~ gamma(0.05, 10), initial value = 1
- G-T substitutions ~ gamma(0.05, 10), initial value = 1
- Base frequences ~ uniform(0, 1), initial value = 0.25
- Gamma shape parameter ~ exponential(0.5), initial value = 0.5
- Proportion of invariant sites ~ uniform(0, 1), initial value = 0.5
- Relative rates among partitions ~ uniform(0, 1E100), initial value = 1
- Birth-death birth rate ~ uniform(0, 1E5), initial value = 0.01
- Birth-death relative death rate ~ uniform(0, 1), initial value = 0.5
- Proportion of taxa sampled from birth-death tree ~ beta(1, 1), initial = 0.01

Previous sensitivity analyses have determined that these priors are sufficiently diffuse to have little influence on the posterior distributions of the parameters. The strict clock rate was fixed at one so that the branch lengths of the phylogenetic trees are scaled as substitutions per site.

#### Supplementary Results

##### Bat species distributions

The distribution of bat species among the sampled locations was highly variable (Fig B). Specifically, there was limited overlap in species among locations and most locations were dominated by one species. Stoliczka’s trident bats (*Aselliscus stoliczkanus*, Hipposideridae), a fulvus roundleaf bat (*Hipposideros fulvus*, Hipposideridae), horshoe bats (*Rhinolophus* sp., Rhinolophidae), and a black-bearded tomb bat (*Taphozous melanopogon*, Emballonuridae) were captured in Wat Tham Phra Cave in Chiang Rai. Sia Cave in Kamphaeng Phet was dominated by intermediate roundleaf bats (*H. larvatus*), with a few great roundleaf bats (*H. armiger*) and a single horshoe bat (*Rhinolophus* sp.). The bat cave in Khon Kaen produced mostly *H. armiger*, but also some wrinkle-lipped free-tailed bats (*Chaerephon plicatus*, Molossidae) and a single roundleaf bat (*Hipposideros* sp.). Khao Chakan Cave in Sa Kaeo produced only *C. plicatus*. Not all samples produced adequate blood samples for culturing, so the total counts per species were slightly different between Fig B and Table A.

**Fig B. Counts of bat species captured in four provinces of Thailand.** Species were identified as close to the species level as possible. Three individuals were only identified to the genus level (*Hipposideros* and *Rhinolophus* sp.).

##### *Bartonella* prevalence and distribution of genogroups

There was some variation in *Bartonella* spp. prevalence among bat species and capture locations (Table A). In Chiang Rai, no bartonellae were cultured from *Rhinolophus* sp. or *A. stoliczkanus*, however the one *H. fulvus* individual captured was positive for *Bartonella* genogroup H3 according to its *gltA* sequence (Fig 1). In Kamphaeng Phet, *H. armiger* and *Rhinolophus* sp. showed no infection while 52.2% (12/23) of *H. larvatus* individuals and the single *T. melanopogon* individual were harboring bartonellae. *H. larvatus* individuals were found to be carrying *Bartonella* genogroups H1, H2, and H3 and the *T. melanopogon* was carrying *Bartonella* genogroup Tm. In Sa Kaeo, 41.5% (17/41) of captured *C. plicatus* individuals were carrying bartonellae; *Bartonella* genogroups Cp1, Cp2, and Cp3 were found in these bats. In Khon Kaen, *C. plicatus* and *Hipposideros* sp. individuals were negative for bartonellae, while 21.4% (3/14) of captured *H. armiger* individuals were infected with *Bartonella* genogroups H1 and H3. Despite the observed variation in *Bartonella* spp. prevalence, the differences among locations (\(\chi^{2}\) = 4.94, p-value = 0.18) and species (\(\chi^{2}\) = 12.5, p-value = 0.09) were not statistically significant, most likely due to the small sample size of bat species from each location. Larger sample sizes will be required to find significant differences among bat species and locations.

The counts of genogroups are larger than to total number of infected individuals due to the presence of multiple cultures originating from the same individual bat (Table A). The *T. melanopogon* individual (KP283) from Kamphaeng Phet produced two cultures (KP283a and KP283b), both of which were *Bartonella* genogroup Tm according to *gltA* (Fig 1); due to this similarity, only culture KP283b was chosen for multi-locus characterization. One *C. plicatus* individual (SK198) from Sa Kaeo produced two cultures (SK198a and SK198b) which were both *Bartonella* genogroup Cp3, so only culture SK198a was chosen for multi-locus characterization. One *H. armiger* individual (KK200) produced two cultures (KK200a and KK200b) which were both *Bartonella* genogroup H1, so only culture KK200a was characterized with other loci. Two *H. larvatus* individuals from Kamphaeng Phet (KP216 and KP268) produced two cultures each of genogroup H1 (KP216a, KP216b, KP268a, and KP268b), so only KP216a and KP268a was further characterized. Another *H. larvatus* bat from Kamphaeng Phet (KP287) produced multiple cultures, but only one (KP287a) was confirmed as a *Bartonella* species. Finally, *H. larvatus* KP293 from Kamphaeng Phet produced one culture (KP293a) which was genogroup H1 and a second culture (KP293b) which was genogroup H2. Only culture (KP293a) was chosen for further characterization since KP293b was so similar to culture KP277 (genogroup H2). Additional isolates from *C. plicatus* in Sa Kaeo (SK137), *H. armiger* in Khon Kaen (KK209), *H. larvatus* in Kamphaeng Phet (KP239, KP261, KP263, and KP274) were left out of the multi-locus characterization because they represented genogroups (Cp3, H1, and H3) which had already been detected. In summary, from the total of 42 isolates obtained from the bats, we narrowed the number of isolates selected for multi-locus characterization to 30 which were representative of the 7 *Bartonella* genogroups identified by *gltA* sequences (Fig 1). As noted in the main text, *Bartonella* genogroups segregated strictly among bat genera (Table A): genogroups Cp1-3 were found only in *C. plicatus*, genogroups H1-3 were found only in *Hipposideros* spp., and genogroup Tm was only found in *T. melanopogon*. However, it should be noted that the small sample size and limited species overlap among locations in our study precludes us from accurately estimating the rate at which these bat species may share *Bartonella* genogroups, especially if exchanges are rare.

**Table A. Counts of bat species captured and *Bartonella* genogroups cultured in four provinces of Thailand.** Total infected bats are recorded for each location. The counts of *Bartonella* genogroups for some species are larger than the number of infected bats because multiple cultures were isolated from some individuals, including some coinfections of multiple genogroups. Exact 95% binomial confidence intervals for prevalence were estimated using the Clopper-Pearson method.

| Province | Species | Family | Count | Positive | Prevalence (%) | 95% confidence interval (%) | *gltA* genogroups (count) |
| --- | --- | --- | --- | --- | --- | --- | --- |
| Chiang Rai | *Aselliscus stoliczkanus* | Hipposideridae | 3 | 0 | 0 | [0, 70.8] |  |
|  | *Hipposideros fulvus* | Hipposideridae | 1 | 1 | 100 | [2.5, 100] | H3 (1) |
|  | *Rhinolophus* sp. | Rhinolphidae | 1 | 0 | 0 | [0, 97.5] |  |
|  |  |  | 5 | 1 | 20 | [0.5, 71.6] |  |
| Kamphaeng Phet | *Hipposideros armiger* | Hipposideridae | 4 | 0 | 0 | [0, 60.2] |  |
|  | *Hipposideros larvatus* | Hipposideridae | 23 | 12 | 52.2 | [30.6, 73.2] | H1 (10), H2 (2), H3 (3) |
|  | *Rhinolophus* sp. | Rhinolophidae | 1 | 0 | 0 | [0, 97.5] |  |
|  | *Taphozous melanopogon* | Emballonuridae | 1 | 1 | 100 | [2.5, 100] | Tm (2) |
|  |  |  | 29 | 13 | 44.8 | [26.4, 64.3] |  |
| Sa Kaeo | *Chaerephon plicatus* | Molossidae | 41 | 17 | 41.5 | [26.3, 57.9] | Cp1 (10), Cp2 (2), Cp3 (6) |
| Khon Kaen | *Chaerephon plicatus* | Molossidae | 3 | 0 | 0 | [0, 70.8] |  |
|  | *Hipposideros armiger* | Hipposideridae | 14 | 3 | 21.4 | [4.7, 50.8] | H1 (3), H3 (1) |
|  | *Hipposideros* sp. | Hipposideridae | 1 | 0 | 0 | [0, 97.5] |  |
|  |  |  | 18 | 3 | 16.7 | [3.6, 41.4] |  |
| **Total** |  |  | 93 | 34 | 36.6 | [26.8, 47.2] |  |

##### Separate gene trees for MLST loci

Maximum likelihood (ML) phylogenies generated for each locus (*ftsZ*, *gltA*, *nuoG*, *rpoB*, and ITS) separately using RAxML v8.2.10 (Stamatakis 2014) showed that most cultures clustered into the same genogroups as identified by *gltA* sequences (Figs C-G). The two exceptions were isolates KP174 and KP287a. Isolate KP174 from a *Hipposideros* sp. bat in Kamphaeng Phet was identified as genogroup H1 by all loci except *nuoG* where it clustered with genogroup H3. Isolate KP287a from a *H. larvatus* individual in Kamphaeng Phet was identified as genogroup H1 by all loci except *rpoB* where it clustered with genogroup H2.

**Fig C. Maximum likelihood phylogeny for *ftsZ* sequences from Thai bats.** The phylogeny was created with RAxML using the GTRCAT model with 25 per site rate categories. Node support was estimated with 1000 bootstrap replicates and is indicated by the size and color of circles at each node. Tip labels for recombinant strains are colored red.

**Fig D. Maximum likelihood phylogeny for *gltA* sequences from Thai bats.** The phylogeny was created with RAxML using the GTRCAT model with 25 per site rate categories. Node support was estimated with 1000 bootstrap replicates and is indicated by the size and color of circles at each node. Tip labels for recombinant strains are colored red.

**Fig E. Maximum likelihood phylogeny for *nuoG* sequences from Thai bats.** The phylogeny was created with RAxML using the GTRCAT model with 25 per site rate categories. Node support was estimated with 1000 bootstrap replicates and is indicated by the size and color of circles at each node. Tip labels for recombinant strains are colored red.

**Fig F. Maximum likelihood phylogeny for *rpoB* sequences from Thai bats.** The phylogeny was created with RAxML using the GTRCAT model with 25 per site rate categories. Node support was estimated with 1000 bootstrap replicates and is indicated by the size and color of circles at each node. Tip labels for recombinant strains are colored red.

**Fig G. Maximum likelihood phylogeny for ITS sequences from Thai bats.** The phylogeny was created with RAxML using the GTRCAT model with 25 per site rate categories. Node support was estimated with 1000 bootstrap replicates and is indicated by the size and color of circles at each node. Tip labels for recombinant strains are colored red.

#### Sequence References

Sequences representing reference strains of *Bartonella* species and also previously identified *Bartonella* strains from bats or other mammal hosts were used to reconstruct the *gltA* phylogeny (Fig 1) and multi-locus phylogeny (Fig 2) in the main text. GenBank accession numbers for these sequences are listed in Table B.

**Table B. GenBank accession numbers for *ftsZ*, *gltA*, *nuoG*, *rpoB*, and ITS sequences from *Bartonella* reference strains and *Bartonella* genogroups from mammal hosts.** Blank references for some strains indicate that no sequence for that locus has been listed on GenBank. References for some *Bartonella* strains from bats and other mammals are listed.

| Species | Strain | ftsZ | gltA | nuoG | rpoB | ITS | Reference |
| --- | --- | --- | --- | --- | --- | --- | --- |
| *Bartonella acomydis* | KS2-1 | AB602545 | AB444979 |  | AB529942 | AB602563 |  |
| *Bartonella alsatica* | IBS382 | NZ\_JH725020 | NZ\_JH725020 | NZ\_JH725020 | NZ\_JH725020 | AF312506 |  |
| *Bartonella ancashensis* | 20.00 | NZ\_CP010401 | NZ\_CP010401 | NZ\_CP010401 | NZ\_CP010401 | KP720644 |  |
| *Bartonella antechini* | MU1-F19 | GU168958 | GU168962 |  | GU168961 | GU168959 |  |
| *Bartonella apis* | PEB0122 | NZ\_LXYU00000000 | NZ\_LXYU00000000 | NZ\_LXYU00000000 | NZ\_LXYU00000000 | NZ\_LXYU00000000 |  |
| *Bartonella australis* | Aust-NH1 | NC\_020300 | NC\_020300 | NC\_020300 | NC\_020300 | DQ538396 |  |
| *Bartonella bacilliformis* | Ver097 | NZ\_KL503805 | NZ\_KL503803 | NZ\_KL503804 | NZ\_KL503803 | DQ200883 |  |
| *Bartonella bandicootii* | BA1 | HQ444163 | HQ444165 |  | HQ444164 | HQ444162 |  |
| *Bartonella birtlesii* | IBS325 | NZ\_CM001557 | NZ\_CM001557 | NZ\_CM001557 | NZ\_CM001557 | AY116640 |  |
| *Bartonella bovis* | 91-4 | NZ\_CM001844 | NZ\_CM001844 | NZ\_CM001844 | NZ\_CM001844 | KM371094 |  |
| *Bartonella callosciuri* | BR1-1 | AB602541 | AB444977 |  | AB529929 | AB602559 |  |
| *Bartonella capreoli* | IBS193 | AB290192 | AF293392 |  | AB290188 | AB498009 |  |
| *Bartonella chomelii* | A828 | KM215688 | KM215690 | KM215699 | KM215705 | KM215713 |  |
| *Bartonella clarridgeiae* | ATCC51734 | NZ\_JADC01000017 | NZ\_JADC01000010 | NZ\_JADC01000010 | NZ\_JADC01000010 | AF312497 |  |
| *Bartonella coopersplainsensis* | AUST-NH20 | EU111781 | EU111803 |  | EU111792 | EU111770 |  |
| *Bartonella davousti* | BvS14 | KU859898 | KU859878 |  | KU859888 | KU859908 |  |
| *Bartonella doshiae* | NCTC12862 | NZ\_JH725095 | NZ\_JH725094 | NZ\_JH725094 | NZ\_JH725094 | AF442954 |  |
| *Bartonella elizabethae* | F9251 | NZ\_JH725034 | NZ\_JH725033 | NZ\_JH725033 | NZ\_JH725033 | L35103 |  |
| *Bartonella florenciae* | R4 | NZ\_HE997451 | NZ\_HE997451 | NZ\_HE997451 | NZ\_HE997451 | HM622140 |  |
| *Bartonella grahamii* | as4aup | NC\_012846 | NC\_012846 | NC\_012846 | NC\_012846 | JN810847 |  |
| *Bartonella henselae* | Houston-1 | NC\_005956 | NC\_005956 | NC\_005956 | NC\_005956 | L35101 |  |
| *Bartonella jaculi* | OY2-1 | AB602539 | AB444975 |  | AB529934 | AB602557 |  |
| *Bartonella japonica* | Fuji\_18-1 | AB440633 | AB242289 |  | AB242288 | AB498007 |  |
| *Bartonella koehlerae* | C-29 | NZ\_KL407334 | NZ\_KL407334 | NZ\_KL407334 | NZ\_KL407334 | AF312490 |  |
| *Bartonella lascolai* | C102 | KP715476 | KP715477 |  |  | KP715478 | (Davoust et al. 2016) |
| *Bartonella mastomydis* | 205-2 |  | KR107234 |  | KP997021 | KT895985 |  |
| *Bartonella mayotimonensis* | EYL-2008 | FJ376734 | FJ376732 |  | FJ376736 | FJ376735 | (E. Y. Lin et al. 2010) |
| *Bartonella melophagi* | K-2C | NZ\_JH725081 | NZ\_JH725083 | NZ\_JH725082 | NZ\_JH725083 | JF834886 |  |
| *Bartonella pachyuromydis* | FN15-2 | AB602543 | AB444978 |  | AB602555 | AB602561 |  |
| *Bartonella phoceensis* | 16120 | AY515135 | AY515126 |  | AY515132 | AY515123 |  |
| *Bartonella queenslandensis* | AUST-NH15 | NZ\_HE997987 | NZ\_HE998003 | NZ\_HE997985 | NZ\_HE998003 | EU111769 |  |
| *Bartonella quintana* | Toulouse | NC\_005955 | NC\_005955 | NC\_005955 | NC\_005955 | L35100 |  |
| *Bartonella rattaustraliani* | AUST-NH4 | NZ\_CALW02000009 | NZ\_CALW02000061 | NZ\_CALW02000050 | NZ\_CALW02000063 | EU111760 |  |
| *Bartonella rattimassiliensis* | 15908 | NZ\_JH725069 | NZ\_JH725064 | NZ\_JH725064 | NZ\_JH725065 | AY515121 |  |
| *Bartonella rochalimae* | ATCCBAA-1498 | NZ\_KL407337 | NZ\_KL407337 | NZ\_KL407337 | NZ\_KL407337 | DQ683199 |  |
| *Bartonella rolaini* | C65 | KP715472 | KP715473 |  | KP715475 | KP715474 | (Davoust et al. 2016) |
| *Bartonella rondoniensis* | clone-1-2 | KX377404 |  |  | KX377405 |  |  |
| *Bartonella rudakovii* | St490 | EF682092 | EF682090 |  | EF682088 | EF682087 |  |
| *Bartonella schoenbuchensis* | R1 | FN645509 | FN645507 | FN645509 | FN645507 | EF418055 |  |
| \*Bartonella senegalensis | OSO2 | NZ\_HE997541 | NZ\_HE997540 | NZ\_HE997542 | NZ\_HE997540 | HM636451 |  |
| *Bartonella silvatica* | Fuji\_23-1 | AB440637 | AB242287 |  | AB242292 | AB498008 |  |
| *Bartonella silvicola* | Cul-9 |  | EF616479 |  | EF616477 | EF616480 | unpublished |
| *Bartonella tamiae* | Th239 | NZ\_JH725147 | NZ\_JH725147 | NZ\_JH725147 | NZ\_JH725147 | DQ395180 |  |
| *Bartonella taylorii* | 8TBB | NZ\_JH725051 | NZ\_JH725052 | NZ\_JH725052 | NZ\_JH725052 | AJ269784 |  |
| *Bartonella thailandensis* | Bthai | FJ411481 | FJ411482 |  | FJ411483 | FJ411485 |  |
| *Bartonella tribocorum* | BM1374166 | NZ\_HG969192 | NZ\_HG969192 | NZ\_HG969192 | NZ\_HG969192 | AF312505 |  |
| *Bartonella vinsonii* subsp. *vinsonii* | Baker | AF467757 | Z70015 | EF659944 | AF165997 | L35102 |  |
| *Bartonella washoensis* | Sb944nv | NZ\_JH725024 | NZ\_JH725022 | NZ\_JH725025 | NZ\_JH725022 | AB674253 |  |
| *Bartonella woyliei* | WC1 | HQ444150 | HQ444152 |  | HQ444151 | HQ444149 |  |
| *Brucella abortus* | Biovar-3 | NZ\_DS999886 | NZ\_DS999886 | NZ\_DS999883 | NZ\_DS999886 | X95889 |  |
| *Coleura afra* | C-583 | HQ832883 | HM545136 |  | MF288100 | KM382256 | (M. Y. Kosoy et al. 2010) |
| *Corynorhinus townsendii* | ZAG01 |  | JX416236 |  |  |  | (Morse et al. 2012) |
| Dog | BK11 |  | FJ946847 |  |  |  | (Bai et al. 2010) |
| Dog | BK62 |  | FJ946848 |  |  |  | (Bai et al. 2010) |
| Dog | KK48 |  | FJ946850 |  |  |  | (Bai et al. 2010) |
| Dog | KK49 |  | FJ946851 |  |  |  | (Bai et al. 2010) |
| Dog | KK61 |  | FJ946852 |  |  |  | (Bai et al. 2010) |
| Dog | KK14 |  | FJ946853 |  |  |  | (Bai et al. 2010) |
| Dog | KK20 |  | FJ946854 |  |  |  | (Bai et al. 2010) |
| Dog | KK45 |  | FJ946855 |  |  |  | (Bai et al. 2010) |
| *Eidolon dupreanum* | 1\_CycDub |  | KT751146 |  |  |  | (Wilkinson et al. 2016) |
| *Eidolon dupreanum* | 4\_CycDub |  | KT751148 |  |  |  | (Wilkinson et al. 2016) |
| *Eidolon dupreanum* | 5\_CycDub |  | KT751149 |  |  |  | (Wilkinson et al. 2016) |
| *Eidolon dupreanum* | 6\_CycDub |  | KT751150 |  |  |  | (Wilkinson et al. 2016) |
| *Eidolon dupreanum* | 7\_CycDub |  | KT751151 |  |  |  | (Wilkinson et al. 2016) |
| *Eidolon helvum* | E1-105 | HM363770 | HM363765 | MF288093 | HM363775 | MF288105 | (M. Y. Kosoy et al. 2010) |
| *Eidolon helvum* | E2-114 | HM363771 | HM363766 | MF288094 | HM363776 | MF288106 | (M. Y. Kosoy et al. 2010) |
| *Eidolon helvum* | E3-106 | HM363772 | HM363767 | MF288095 | HM363777 | MF288107 | (M. Y. Kosoy et al. 2010) |
| *Eidolon helvum* | Ew-111 | HM363773 | HM363768 | MF288096 | HM363778 | MF288108 | (M. Y. Kosoy et al. 2010) |
| *Eidolon helvum* | B39301 | KJ999687 | KM030516 | KM030541 | KM215201 | MF288109 | (Bai et al. 2015) |
| *Eidolon helvum* | B40014 | KJ999689 | KM030520 | KM030542 | KM215202 | KM233479 | (Bai et al. 2015) |
| *Eptesicus nilssoni* | 1157-3 | KF003121 | KF003115 |  | KF003118 | KF003117 | (Veikkolainen et al. 2014) |
| *Eptesicus serotinus* | 44722 | KX300199 | KX300200 | KX300202 | KX300203 | MF288110 | (Urushadze et al. 2017) |
| *Harpyionycteris whiteheadi* | JAE1033 |  | JX416239 |  |  |  | (Morse et al. 2012) |
| *Hipposideros armiger* | B095 |  | KP100356 |  |  |  | (Anh et al. 2015) |
| *Hipposideros armiger* | B096 |  | KP100357 |  |  |  | (Anh et al. 2015) |
| *Hipposideros larvatus* | B081 |  | KP100354 |  |  |  | (Anh et al. 2015) |
| *Hipposideros larvatus* | B087 |  | KP100355 |  |  |  | (Anh et al. 2015) |
| *Hipposideros larvatus* | B110 |  | KP100360 |  |  |  | (Anh et al. 2015) |
| *Hipposideros* sp. | Mala15 |  | JX416238 |  |  |  | (Morse et al. 2012) |
| *Hipposideros vittatus* (formerly *H. commersoni*) | H-556 | KM382254 | HM545137 | KM382252 |  | KM382258 | (M. Y. Kosoy et al. 2010) |
| Human | G19 |  | HM116784 |  |  |  | (Podsiadly et al. 2010) |
| Human | G10 |  | HM116785 |  |  |  | (Podsiadly et al. 2010) |
| Human | G14 |  | HM116786 |  |  |  | (Podsiadly et al. 2010) |
| *Megaderma lyra* | B109 |  | KP100359 |  |  |  | (Anh et al. 2015) |
| *Megaderma spasma* | B005 |  | KP100341 |  |  |  | (Anh et al. 2015) |
| *Meriones libycus* | B29881 | KT327035 | KT327028 | KT327037 | KT327041 | JF766260 | (Malania et al. 2016) |
| *Meriones libycus* | B29771 | KT327034 | KT327027 | KT327036 | KT327040 | JF766255 | (Malania et al. 2016) |
| *Miniopterus aelleni* | 11c\_PenLep |  | KT751143 |  |  |  | (Wilkinson et al. 2016) |
| *Miniopterus gleni* | Gr16\_NycSty |  | KT751152 |  |  |  | (Wilkinson et al. 2016) |
| *Miniopterus griveaudi* | Gr20\_PenLep |  | KT751153 |  |  |  | (Wilkinson et al. 2016) |
| *Miniopterus griveaudi* | Gr6\_NycSty |  | KT751155 |  |  |  | (Wilkinson et al. 2016) |
| *Miniopterus minor* | M1-44 | MF288090 | HM545139 | MF288097 | MF288101 | MF288111 | (M. Y. Kosoy et al. 2010) |
| *Miniopterus natalensis* | M3-373 | MF288091 | HM545141 | MF288098 | MF288102 | MF288112 | (M. Y. Kosoy et al. 2010) |
| *Miniopterus schreibersii* | No.05 | JF500495 | JF500511 |  | JF500543 |  | (J.-W. Lin et al. 2012) |
| *Miniopterus schreibersii* | 44530 | KX300175 | KX300183 | KX300185 | KX300186 | MF288113 | (Urushadze et al. 2017) |
| *Miniopterus schreibersii* | 44608 | KY679153 | KX300195 | KX300197 | KX300198 | MF288114 | (Urushadze et al. 2017) |
| *Miniopterus schreibersii* | 44599 | KX300191 | KX300192 | KX300193 | KX300194 | MF288115 | (Urushadze et al. 2017) |
| *Miniopterus schreibersii* | 44593 | KX300187 | KY679154 | KX300190 | KX300188 | MF288116 | (Urushadze et al. 2017) |
| *Miniopterus* sp. | M2-491 |  | HM545140 |  |  |  | (M. Y. Kosoy et al. 2010) |
| *Miniopterus* sp. | 12\_PenLep |  | KT751144 |  |  |  | (Wilkinson et al. 2016) |
| *Miniopterus* sp. | 13b\_PenLep |  | KT751145 |  |  |  | (Wilkinson et al. 2016) |
| *Myotis blythii* | 44731 | KX300139 | KX300140 | KX300142 | KX300143 | MF288117 | (Urushadze et al. 2017) |
| *Myotis blythii* | 44602 | KX300116 | KX300117 | KX300119 | KX300120 | MF288118 | (Urushadze et al. 2017) |
| *Myotis blythii* | 44601 | KX300111 | KX300112 | KX300114 | KX300115 | MF288119 | (Urushadze et al. 2017) |
| *Myotis blythii* | 44622 | KX300121 | KX300123 | KX300125 | KX300122 | MF288120 | (Urushadze et al. 2017) |
| *Myotis blythii* | 44711 | KX300126 | KX300127 | KX300128 | KX300129 | MF288121 | (Urushadze et al. 2017) |
| *Myotis blythii* | 44719 | KX300134 | KX300136 | KX300138 | KX300135 | MF288122 | (Urushadze et al. 2017) |
| *Myotis blythii* | 44715 | KX300130 | KX300131 | KX300132 | KX300133 | MF288123 | (Urushadze et al. 2017) |
| *Myotis blythii* | 44591 | KX300106 | KX300107 | KX300109 | KX300110 | MF288124 | (Urushadze et al. 2017) |
| *Myotis daubentonii* | 1160-1 | KF003128 | KF003122 |  | KF003125 | KF003124 | (Veikkolainen et al. 2014) |
| *Myotis daubentonii* | 2574-1 | KF003135 | KF003129 |  | KF003132 | KF003131 | (Veikkolainen et al. 2014) |
| *Myotis daubentonii* | bat2053\_2034 |  | KR822802 |  |  |  | (Lilley, Veikkolainen, and Pulliainen 2015) |
| *Myotis daubentonii* | M406 |  | AJ871613 |  |  |  | (Concannon et al. 2005) |
| *Myotis emarginatus* | 44617 | KX300148 | KX300149 | KX300151 | KX300152 | MF288125 | (Urushadze et al. 2017) |
| *Myotis emarginatus* | 44724 | KX300153 | KX300154 | KX300156 | KX300157 | MF288126 | (Urushadze et al. 2017) |
| *Myotis emarginatus* | 44544 | KX300176 | KX300145 | KX300144 | KX300147 | MF288127 | (Urushadze et al. 2017) |
| *Myotis fimbriatus* | SD-73 |  | KX655821 |  |  |  | (Han et al. 2017) |
| *Myotis fimbriatus* | SD-74-2 |  | KX655808 |  |  |  | (Han et al. 2017) |
| *Myotis fimbriatus* | SD-72 |  | KX655834 |  |  |  | (Han et al. 2017) |
| *Myotis fimbriatus* | SD-75 |  | KX655822 |  |  |  | (Han et al. 2017) |
| *Myotis fimbriatus* | SD-70 |  | KX655829 |  |  |  | (Han et al. 2017) |
| *Myotis fimbriatus* | SD-78 |  | KX655839 |  |  |  | (Han et al. 2017) |
| *Myotis fimbriatus* | SD-74-1 |  | KX655827 |  |  |  | (Han et al. 2017) |
| *Myotis fimbriatus* | SD-76 |  | KX655843 |  |  |  | (Han et al. 2017) |
| *Myotis fimbriatus* | SD-62 |  | KX655832 |  |  |  | (Han et al. 2017) |
| *Myotis grisescens* | UTK0803 |  | KX807176 |  |  |  | (Lilley et al. 2017) |
| *Myotis grisescens* | UTK1106 |  | KX807175 |  |  |  | (Lilley et al. 2017) |
| *Myotis keaysi* | SJ126 |  | KJ816667 |  |  |  | (Judson, Frank, and Hadly 2015) |
| *Myotis keaysi* | SJ125 |  | KJ816669 |  |  |  | (Judson, Frank, and Hadly 2015) |
| *Myotis keaysi* | SJ132 |  | KJ816689 |  |  |  | (Judson, Frank, and Hadly 2015) |
| *Myotis lucifugus* | DMR02097 |  | KX807171 |  |  |  | (Lilley et al. 2017) |
| *Myotis lucifugus* | DMR02036 |  | KX807174 |  |  |  | (Lilley et al. 2017) |
| *Myotis lucifugus* | DMR02098 |  | KX807173 |  |  |  | (Lilley et al. 2017) |
| *Myotis lucifugus* | DMR02005 |  | KX807178 |  | KX807183 | KX807186 | (Lilley et al. 2017) |
| *Myotis lucifugus* | DMR02028 |  | KX807179 |  | KX807184 | KX807187 | (Lilley et al. 2017) |
| *Myotis lucifugus* | DMR02051 |  | KX807177 |  | KX807182 | KX807185 | (Lilley et al. 2017) |
| *Myotis lucifugus* | DMR02050 |  | KX807172 |  |  |  | (Lilley et al. 2017) |
| *Myotis myotis* | AS048 |  | JQ695836 |  |  |  | unpublished |
| *Myotis myotis* | AS036 |  | JQ695837 |  |  |  | unpublished |
| *Myotis myotis* | AS033 |  | JQ695838 |  |  |  | unpublished |
| *Myotis myotis* | AS025 |  | JQ695839 |  |  |  | unpublished |
| *Myotis myotis* | AS067 |  | JQ695840 |  |  |  | unpublished |
| *Myotis myotis* | AS071 |  | JQ695834 |  |  |  | unpublished |
| *Myotis myotis* | AS050 |  | JQ695835 |  |  |  | unpublished |
| *Myotis mystacinus* | M62 |  | AJ871612 |  |  |  | (Concannon et al. 2005) |
| *Myotis nattererei* | ZAG03 |  | JX416241 |  |  |  | (Morse et al. 2012) |
| *Myotis pequinius* | SD-123 |  | KX655815 |  |  |  | (Han et al. 2017) |
| *Myotis pequinius* | SD-119 |  | KX655825 |  |  |  | (Han et al. 2017) |
| *Myotis pequinius* | SD-120-2 |  | KX655816 |  |  |  | (Han et al. 2017) |
| *Myotis pequinius* | SD-109 |  | KX655842 |  |  |  | (Han et al. 2017) |
| *Myotis pequinius* | SD-142 |  | KX655830 |  |  |  | (Han et al. 2017) |
| *Myotis pequinius* | SD-93 |  | KX655828 |  |  |  | (Han et al. 2017) |
| *Myotis pequinius* | SD-111 |  | KX655841 |  |  |  | (Han et al. 2017) |
| *Myotis pequinius* | SD-91 |  | KX655812 |  |  |  | (Han et al. 2017) |
| *Myotis pequinius* | SD-99 |  | KX655823 |  |  |  | (Han et al. 2017) |
| *Myotis pequinius* | SD-120-1 |  | KX655814 |  |  |  | (Han et al. 2017) |
| *Myotis pequinius* | SD-122 |  | KX655820 |  |  |  | (Han et al. 2017) |
| *Myotis pequinius* | SD-138 |  | KX655836 |  |  |  | (Han et al. 2017) |
| *Myotis pequinius* | SD-96 |  | KX655833 |  |  |  | (Han et al. 2017) |
| *Myotis pequinius* | SD-117 |  | KX655835 |  |  |  | (Han et al. 2017) |
| *Myotis ricketti* | SD-83 |  | KX655811 |  |  |  | (Han et al. 2017) |
| *Myotis* sp. | B32942 |  | JQ071390 |  |  |  | (Bai et al. 2012) |
| *Nyctalus noctula* | M451 |  | AJ871615 |  |  |  | (Concannon et al. 2005) |
| *Pipistrellus pygmaeus* | 44718 | KX300177 | KX300179 | KX300178 | KX300181 | MF288128 | (Urushadze et al. 2017) |
| *Pipistrellus* sp. | M409 |  | AJ871611 |  |  |  | (Concannon et al. 2005) |
| *Pipistrellus* sp. | M207 |  | AJ871614 |  |  |  | (Concannon et al. 2005) |
| *Ptenochirus jagori* | P2874 |  | JX416255 |  |  |  | (Morse et al. 2012) |
| *Pteropus hypomelanus* | E7 |  | JX416257 |  |  |  | (Morse et al. 2012) |
| *Pteropus hypomelanus* | E5 |  | JX416256 |  |  |  | (Morse et al. 2012) |
| *Rattus norvegicus* | 1-1C | FN645499 | FN645496 | FN645496 | FN645496 | EU551157 | (Engel et al. 2011) |
| *Rhinolophus acuminatus* | B003 |  | KP100340 |  |  |  | (Anh et al. 2015) |
| *Rhinolophus acuminatus* | B006 |  | KP100342 |  |  |  | (Anh et al. 2015) |
| *Rhinolophus acuminatus* | B050 |  | KP100345 |  |  |  | (Anh et al. 2015) |
| *Rhinolophus acuminatus* | B055 |  | KP100347 |  |  |  | (Anh et al. 2015) |
| *Rhinolophus acuminatus* | B056 |  | KP100348 |  |  |  | (Anh et al. 2015) |
| *Rhinolophus acuminatus* | B063 |  | KP100349 |  |  |  | (Anh et al. 2015) |
| *Rhinolophus acuminatus* | B064 |  | KP100350 |  |  |  | (Anh et al. 2015) |
| *Rhinolophus acuminatus* | B068 |  | KP100351 |  |  |  | (Anh et al. 2015) |
| *Rhinolophus euryale* | 44528 | KX300105 | KX300158 | KX300160 | KX300161 | MF288129 | (Urushadze et al. 2017) |
| *Rhinolophus ferrumequinum* | SD-3 |  | KX655838 |  |  |  | (Han et al. 2017) |
| *Rhinolophus ferrumequinum* | 44706 | KX300168 | KX300169 | KX300172 | KX300173 | MF288130 | (Urushadze et al. 2017) |
| *Rhinolophus ferrumequinum* | 44658 | KX300174 | KX300165 | KX300167 | KX300170 | MF288131 | (Urushadze et al. 2017) |
| *Rhinolophus ferrumequinum* | 44552 | KX300182 | KY679155 | KX300163 | KX300164 | MF288132 | (Urushadze et al. 2017) |
| *Rhinolophus pearsoni* | 05\_01\_07 |  | JX416252 |  |  |  | (Morse et al. 2012) |
| *Rhinolophus pusillus* | SD-19 |  | KX655824 |  |  |  | (Han et al. 2017) |
| *Rhinolophus pusillus* | SD-16 |  | KX655818 |  |  |  | (Han et al. 2017) |
| *Rhinolophus sinicus* | B049 |  | KP100344 |  |  |  | (Anh et al. 2015) |
| *Rousettus aegyptiacus* | R-191 | HM363769 | HM363764 | KM387321 | HM363774 | KM382255 | (M. Y. Kosoy et al. 2010) |
| *Scotophilus marovaza* | SC1\_Basilia |  | KT751157 |  |  |  | (Wilkinson et al. 2016) |
| *Scotophilus robustus* | 1b\_Basilia |  | KT751147 |  |  |  | (Wilkinson et al. 2016) |
| *Sorex araneus* | DB5-6 | NZ\_JH725120 | NZ\_JH725114 | NZ\_JH725119 | NZ\_JH725114 |  | unpublished |
| *Tamiasciurus hudsonicus* | AR-15-3 | FN645482 | FN645480 | FN645479 | FN645480 |  | (Engel et al. 2011) |
| *Triaenops persicus* | T-837 | KM382253 | HM545138 | KM382251 | MF288104 | KM382257 | (M. Y. Kosoy et al. 2010) |
| *Tylonycteris* sp. | Mala11 |  | JX416246 |  |  |  | (Morse et al. 2012) |

#### References

Anh, Pham Hong, Nguyen Van Cuong, Nguyen Truong Son, Ngo Tri Tue, Michael Y Kosoy, Mark E J Woolhouse, Stephen Baker, et al. 2015. “Diversity of Bartonella spp. in bats, southern Vietnam.” *Emerging Infectious Diseases* 21 (7): 1266–7. doi:10.3201/eid2107.141760.

Bai, Ying, David T S Hayman, Clifton D McKee, and Michael Y Kosoy. 2015. “Classification of Bartonella strains associated with straw-colored fruit bats (Eidolon helvum) across Africa using a multi-locus sequence typing platform.” *PLOS Neglected Tropical Diseases* 9 (1): e0003478. doi:10.1371/journal.pntd.0003478.

Bai, Ying, Michael Y Kosoy, Sumalee Boonmar, Pongpun Sawatwong, Somboon Sangmaneedet, and Leonard F Peruski. 2010. “Enrichment culture and molecular identification of diverse Bartonella species in stray dogs.” *Veterinary Microbiology* 146 (3-4). Elsevier B.V.: 314–19. doi:10.1016/j.vetmic.2010.05.017.

Bai, Ying, Sergio Recuenco, Amy T Gilbert, Lynn M Osikowicz, Jorge Gomez, Charles E Rupprecht, and Michael Y Kosoy. 2012. “Prevalence and diversity of Bartonella spp. in bats in Peru.” *American Journal of Tropical Medicine and Hygiene* 87 (3): 518–23. doi:10.4269/ajtmh.2012.12-0097.

Concannon, R, K Wynn-Owen, V R Simpson, and Richard J Birtles. 2005. “Molecular characterization of haemoparasites infecting bats (Microchiroptera) in Cornwall, UK.” *Parasitology* 131 (04): 489–96. doi:10.1017/S0031182005008097.

Davoust, Bernard, Jean-Lou Marié, Mustapha Dahmani, Jean-Michel Berenger, Jean-Michel Bompar, Denis Blanchet, Marie Cheuret, Didier Raoult, and Oleg Mediannikov. 2016. “Evidence of Bartonella spp. in blood and ticks (Ornithodoros hasei) of bats, in French Guiana.” *Vector-Borne and Zoonotic Diseases* 16 (8): 516–19. doi:10.1089/vbz.2015.1918.

Engel, Philipp, Walter Salzburger, Marius Liesch, Chao Chin Chang, Soichi Maruyama, Christa Lanz, Alexandra Calteau, et al. 2011. “Parallel evolution of a type IV secretion system in radiating lineages of the host-restricted bacterial pathogen bartonella.” *PLOS Genetics* 7 (2): e1001296. doi:10.1371/journal.pgen.1001296.

Han, Hui-Ju, Hong-ling Wen, Li Zhao, Jian-Wei Liu, Li-Mei Luo, Chuan-Min Zhou, Xiang-Rong Qin, Ye-Lei Zhu, Xue-Xing Zheng, and Xue-Jie Yu. 2017. “Novel Bartonella species in insectivorous bats, northern China.” Edited by Ulrike Gertrud Munderloh. *PLOS ONE* 12 (1): e0167915. doi:10.1371/journal.pone.0167915.

Judson, S D, H K Frank, and E A Hadly. 2015. “Bartonellae are prevalent and diverse in Costa Rican bats and bat flies.” *Zoonoses and Public Health* 62 (8): 609–17. doi:10.1111/zph.12188.

Kosoy, Michael Y, Ying Bai, Tarah Lynch, Ivan V Kuzmin, Michael Niezgoda, Richard Franka, Bernard Agwanda, Robert F Breiman, and Charles E Rupprecht. 2010. “Bartonella spp. in bats, Kenya.” *Emerging Infectious Diseases* 16 (12). Centers for Disease Control; Prevention, Fort Collins, Colorado 80521, USA. mck3@cdc.gov: 1875–81. doi:10.3201/eid1612.100601.

Kosoy, Michael Y, Russell L Regnery, Theodore Tzianabos, Eric L Marston, Dana C Jones, Douglas Green, Gary O Maupin, James G Olson, and James E Childs. 1997. “Distribution, diversity, and host specificity of Bartonella in rodents from the Southeastern United States.” *American Journal of Tropical Medicine and Hygiene* 57 (5): 578–88. http://www.ncbi.nlm.nih.gov/pubmed/9392599.

Kuzmin, Ivan V, Michael Niezgoda, Richard Franka, Bernard Agwanda, Wanda Markotter, Janet C Beagley, Olga Y Urazova, Robert F Breiman, and Charles E Rupprecht. 2008. “Lagos bat virus in Kenya.” *Journal of Clinical Microbiology* 46 (4): 1451–61. doi:10.1128/JCM.00016-08.

Lilley, Thomas M, Ville Veikkolainen, and Arto T Pulliainen. 2015. “Molecular detection of Candidatus Bartonella hemsundetiensis in bats.” *Vector-Borne and Zoonotic Diseases* 15 (11): 706–8. doi:10.1089/vbz.2015.1783.

Lilley, Thomas M, Cali A Wilson, Riley F Bernard, Emma V Willcox, Eero J Vesterinen, Quinn M R Webber, Laura Kurpiers, et al. 2017. “Molecular detection of Candidatus Bartonella mayotimonensis in North American bats.” *Vector-Borne and Zoonotic Diseases* XX (Xx): vbz.2016.2080. doi:10.1089/vbz.2016.2080.

Lin, Eleanor Y, Constantine Tsigrelis, Larry M Baddour, Hubert Lepidi, Jean-Marc Rolain, Robin Patel, and Didier Raoult. 2010. “Candidatus Bartonella mayotimonensis and endocarditis.” *Emerging Infectious Diseases* 16 (3): 500–503. doi:10.3201/eid1603.081673.

Lin, Jen-Wei, Yuan-Man Hsu, Bruno B Chomel, Liang-Kong Lin, Jai-Chyi Pei, Sheng-Hai Wu, and Chao-Chin Chang. 2012. “Identification of novel Bartonella spp. in bats and evidence of Asian gray shrew as a new potential reservoir of Bartonella.” *Veterinary Microbiology* 156 (1-2). Elsevier B.V.: 119–26. doi:10.1016/j.vetmic.2011.09.031.

Malania, Lile, Ying Bai, Lynn M Osikowicz, Nikoloz Tsertsvadze, Guram Katsitadze, Paata Imnadze, and Michael Kosoy. 2016. “Prevalence and diversity of Bartonella species in rodents from Georgia (Caucasus).” *American Journal of Tropical Medicine and Hygiene* 95 (2): 466–71. doi:10.4269/ajtmh.16-0041.

Morse, Solon F, Kevin J Olival, Michael Y Kosoy, Sarah A Billeter, Bruce D Patterson, Carl W Dick, and Katharina Dittmar. 2012. “Global distribution and genetic diversity of Bartonella in bat flies (Hippoboscoidea, Streblidae, Nycteribiidae).” *Infection, Genetics and Evolution* 12 (8). Elsevier B.V.: 1717–23. doi:10.1016/j.meegid.2012.06.009.

Podsiadly, E, T Chmielewski, G Karbowiak, E Kedra, and S Tylewska-Wierzbanowska. 2010. “The occurrence of spotted fever rickettsioses and other tick-borne infections in forest workers in Poland.” *Vector-Borne and Zoonotic Diseases* 11 (7): 985–89. doi:10.1089/vbz.2010.0080.

Stamatakis, Alexandros. 2014. “RAxML version 8: a tool for phylogenetic analysis and post-analysis of large phylogenies.” *Bioinformatics* 30 (9): 1312–3. doi:10.1093/bioinformatics/btu033.

Urushadze, Lela, Ying Bai, Lynn M Osikowicz, Clifton D McKee, Andrei Kandaurov, Ivan V Kuzmin, Ketevan Sidamonidze, Davit Putkaradze, Paata Imnadze, and Michael Y Kosoy. 2017. “Prevalence, diversity, and host associations of Bartonella strains in bats from Georgia (Caucasus).” *PLOS Neglected Tropical Diseases* 11 (4): e0005428. https://doi.org/10.1371/journal.pntd.0005428{\%}0A.

Veikkolainen, Ville, Eero J Vesterinen, Thomas M Lilley, and Arto T Pulliainen. 2014. “Bats as reservoir hosts of human bacterial pathogen, Bartonella mayotimonensis.” *Emerging Infectious Diseases* 20 (6): 960–67. doi:10.3201/eid2006.130956.

Wilkinson, David A, Olivier Duron, Colette Cordonin, Yann Gomard, Beza Ramasindrazana, Patrick Mavingui, Steven M Goodman, and Pablo Tortosa. 2016. “The bacteriome of bat flies (Nycteribiidae) from the Malagasy region: a community shaped by host ecology, bacterial transmission mode, and host-vector specificity.” *Applied and Environmental Microbiology* 82 (6): 1778–88. doi:10.1128/AEM.03505-15.
